# Drice restrains Diap2-mediated inflammatory signalling and intestinal inflammation

**DOI:** 10.1038/s41418-021-00832-w

**Published:** 2021-07-14

**Authors:** Christa Kietz, Aravind K. Mohan, Vilma Pollari, Ida-Emma Tuominen, Paulo S. Ribeiro, Pascal Meier, Annika Meinander

**Affiliations:** 1 Faculty of Science and Engineering, Cell Biology, Åbo Akademi University, BioCity, Turku, Finland; 2 Centre for Tumour Biology, Barts Cancer Institute, Queen Mary University of London, London, UK; 3 The Breast Cancer Now Toby Robins Research Centre, The Institute of Cancer Research, London, UK

**Keywords:** Ubiquitins, Gene regulation, Antimicrobial responses, Chronic inflammation, Signal transduction

## Abstract

The *Drosophila* IAP protein, Diap2, is a key mediator of NF-κB signalling and innate immune responses. Diap2 is required for both local immune activation, taking place in the epithelial cells of the gut and trachea, and for mounting systemic immune responses in the cells of the fat body. We have found that transgenic expression of Diap2 leads to a spontaneous induction of NF-κB target genes, inducing chronic inflammation in the *Drosophila* midgut, but not in the fat body. Drice is a *Drosophila* effector caspase known to interact and form a stable complex with Diap2. We have found that this complex formation induces its subsequent degradation, thereby regulating the amount of Diap2 driving NF-κB signalling in the intestine. Concordantly, loss of Drice activity leads to accumulation of Diap2 and to chronic intestinal inflammation. Interestingly, Drice does not interfere with pathogen-induced signalling, suggesting that it protects from immune responses induced by resident microbes. Accordingly, no inflammation was detected in transgenic Diap2 flies and Drice-mutant flies reared in axenic conditions. Hence, we show that Drice, by restraining Diap2, halts unwanted inflammatory signalling in the intestine.

## Introduction

Innate immune responses are initiated by pattern recognition receptors (PRRs) that recognize pathogen-associated molecular patterns and danger-associated molecular patterns. Activation of PRRs leads to the induction of microbicidal and pro-inflammatory responses, and culminates in elimination of the activating molecule and subsequent return to cellular homeostasis [1]. Proper regulation of inflammatory signalling is crucial as de-regulation at any step can be detrimental for the organism. One of the key players in the inflammatory response is the NF-κB family of transcription factors that regulate the expression of numerous inflammatory genes. Constitutively active NF-κB signalling is characteristic of chronic inflammation and increased NF-κB activity has been connected to irritable bowel diseases, such as ulcerative colitis and Crohn’s disease [2, 3].

Inhibitor of apoptosis proteins (IAPs) influence ubiquitin-dependent pathways that modulate innate immune signalling by activation of NF-κB [4]. IAPs were first identified in insect baculoviruses as potent inhibitors of cell death [5, 6] and have subsequently been identified in both vertebrates and invertebrates. Cellular and viral IAPs are characterized by the presence of one or more caspase-binding Baculovirus inhibitor of apoptosis protein repeat (BIR) domains that are essential for their anti-apoptotic properties [6, 7], as well as a Really interesting new gene (RING) domain, providing them with E3 ligase activity [8]. *Drosophila* carries two *bona fide* IAP genes, *Drosophila* IAP (Diap) 1 and 2 [9]. Diap1 functions mainly as a suppressor of cell death, whereas Diap2, although also able to decrease the apoptotic threshold of the cell, has its main function in inflammatory signalling [10–13]. The most extensively studied mammalian IAPs, i.e. cellular IAP1/2 and X-chromosome-linked IAP (XIAP), and *Drosophila* Diap2 also harbour a Ubiquitin-associated (UBA) domain enabling them to interact with poly-ubiquitin chains [14, 15].

In contrast to mammals, *Drosophila* relies solely on an innate immune defence when combating pathogenic infections. One of the key components of the fly’s immune system is the production and secretion of antimicrobial peptides (AMPs). AMP production is regulated by two NF-κB pathways, namely, the Toll pathway and the Immune deficiency (Imd) pathway [16]. Upon a gram-negative or gram-positive systemic infection, the Imd and Toll pathways, respectively, activate the production of AMPs in the fat body [17, 18]. However, during local immune responses in the gut, the Imd pathway solely controls the generation of AMPs [19, 20]. The Imd pathway is activated by PGRP-LCx receptors recognizing diaminopimelate (DAP)-type peptidoglycans, which are components of the cell wall of gram-negative bacteria [21–23]. Activation of PGRP-LCx leads to the recruitment of the adaptor proteins Imd and dFadd, and the initiator caspase Dredd [24]. Dredd-mediated cleavage of Imd exposes a conserved IAP-binding motif that recruits Diap2 to the complex, stimulating Diap2-mediated K63-linked ubiquitination (K63-Ub) of Imd, Dredd and the IKKγ Kenny [25–27]. Ubiquitination of Dredd is needed for cleavage and nuclear localization of the NF-κB protein Relish [26, 28], whereas the ubiquitination of Imd has been suggested to recruit the *Drosophila* mitogen-activated protein kinase kinase kinase dTak1 and the Relish kinase complex Ird5/Kenny to the Imd-signalling complex [29].

Vertebrate and invertebrate organisms are in continuous contact with a diverse array of resident microorganisms [30]. One key interface for host–microbe interactions, in both humans and *Drosophila*, is the epithelial layer of the gut [30, 31]. In addition to being a platform for beneficial host-microbe interactions, the gut epithelium serves as the first line of defence against pathogens entering the body. In order for the organism to mount an efficient immune response against pathogenic bacteria, and simultaneously allow commensal bacteria to interact with the host, inflammatory signalling needs to be carefully regulated. Here, we report a novel role of the *Drosophila* caspase-3 homologue Drice as a negative regulator of the Imd pathway in the intestinal epithelium. Our results demonstrate that Drice restrains inflammatory signalling induced by commensal bacteria in the fly intestine by forming a complex with Diap2. This triggers proteasomal degradation of the Drice-Diap2 complex. We also show that transgenic expression of Diap2 leads to chronic inflammation only in the presence of commensal bacteria, which elucidates the need of Diap2 to be regulated in microbiotic environments.

## Material and methods

### Fly husbandry, treatments and strains

*Drosophila melanogaster* was maintained at 25 °C with a 12-h light-dark cycle on Nutri-fly BF (Dutscher Scientific, Essex, UK) food. The *Canton*^*S*^ strain was used as wild type. In experiments with flies expressing genes under the UAS-Gal4 system, the Gal4 driver line was used as internal control. The driver lines *DaughterlessGal4* (*DaGal4*) and *NP1-Gal4*, the *Diptericin-LacZ* reporter line, and *Diap2*^*7c*^ mutants and transgenes were provided by Dr. François Leulier. The *UbiquitousGal4* (*UbiGal4*) strain was provided by Dr. Ville Hietakangas, and *Drice*^*17*^ mutants by Prof. Andreas Bergmann. *PGRP-LCΔ5* (stock #36323) and *UAS-p35* (stock #5073) strains were obtained from Bloomington *Drosophila* stock centre and *Drice-RNAi* (stock #28064) flies were from the Vienna *Drosophila* Resource Centre. V5-tagged *Drice*^*WT*^ and *Drice*^*C211A*^, as well as untagged *Diap2*^*WT*^ and *Diap2*^*Δ100*^ were cloned into pUAST, and transgenic flies were generated by BestGene Inc. Axenic flies were reared germ-free according to the previously published protocol [32]. In short, fly embryos were de-chorionated using 2% active hypochlorite, and washed twice in 70% ethanol and sterile H_2_O. After removal of the chorion, eggs were placed in autoclaved food and left to develop in a sterile environment. The hatched flies were confirmed to be axenic by 16S PCR and by growing fly homogenates on Luria Bertani (LB) plates and checking for bacterial growth. Inhibitor treatments in flies were done by feeding female adult flies for 16 h with 50 µM MG-132 (Sigma, St. Louis, Missouri, USA) or 50 µM Z-DEVD-FMK (BD Pharmingen, Franklin Lakes, New Jersey, USA) diluted in a 1:1 solution of LB media and 5% sucrose after a 2-h starvation.

### Bacterial strains, infection and survival experiments

The gram-negative bacteria *Erwinia carotovora carotovora 15* (*Ecc15*) was kindly provided by Dr. François Leulier and the *Escherichia coli* (*E. coli*) Top10 strain was purchased from Thermo Fisher Scientific (Waltham, Massachusetts, USA). *Ecc15* used for septic infection was cultivated in LB media at 29 °C for 16-18 h on agitation and concentrated (optical density of 200). To induce septic injury, adult flies were pricked in the lateral thorax with a needle previously dipped in concentrated *Ecc15* solution. For quantitative PCR (qPCR) 10 adult flies and for TUBE pulldowns 20 adult flies, were incubated 5 h at 25 °C after infection. For survival assays at least 20 adult flies were counted at indicated time points after infection. Infection experiments were excluded if more than 25% of the negative control strains survived bacterial infection or if AMP gene expression was significantly enhanced in these flies. In these cases, the bacterial potency was considered too low. Survival experiments in which wild-type flies survived to a lesser extent than 75%, were also excluded. These criteria were pre-established. For bacterial colony count, *E. coli* was transformed with pMT/Flag-His-amp and cultivated in ampicillin-containing LB medium at 37 °C for 16-18 h on agitation and concentrated by centrifugation (optical density of 100). After a 2-h starvation, adult flies were fed for 24 h with a 1:1 solution of transformed *E. coli* in 5% sucrose at 25 °C. Four flies were cleaned with ethanol and sterile H_2_O, and homogenised in 300 µl PBS. Samples were diluted 1:1000 and plated on LB agar plates containing 50 µg/ml ampicillin and incubated 24 h at 37 °C, where after the colonies were counted.

### Cell culture and transfection of *Drosophila* S2 cells

*Drosophila* Schneider S2 cells (Invitrogen, Waltham, Massachusetts, USA) were grown at 25 °C using Schneider’s cell medium supplemented with 10% fetal bovine serum, 1% l-glutamine and 0.5% penicillin/streptomycin. S2 cells were transfected with indicated constructs using Effectene transfection reagent (QIAGEN, Hilden, Germany) according to the manufacturer’s instructions. For qPCR, western blotting and for GST-pulldown assays, respectively, 2 × 10^6^ and 0.7 × 10^7^ cells were seeded prior to transfection and the expression of pMT plasmids was induced with 500 µM CuSO_4_ 16 h before lysis. For western blotting, transfected cells were harvested in ice cold PBS and lysed in Laemmli sample buffer. The caspase inhibitor Z-DEVD-FMK was used at 20 µM, 16 h before lysis.

### Plasmids and antibodies

The plasmids pMT/V5His and pAc5/V5His (Invitrogen) were used as backbones for tag insertions and removals, and for subcloning of the constructs pMT/FlagHis, pMT/HAFlag, pAc5/Diap2, pMT/Dredd-V5His, pMT/Dredd-HAFlag, pMT/PGRP-LCx-Myc, pMT/Kenny-HA, pMT/Drice and pMT/ALG(p20/p10)Drice-V5Flag. The point mutation Drice^C211A^ was made by site-directed mutagenesis (Agilent Technologies, Santa Clara, California, USA or Stratagene, San Diego, California, USA). GST-TUBE (tandem ubiquitin entity) was provided by Dr. Mads Gyrd-Hansen. The following antibodies were used: α-K63 (clone Apu3, #05-1308, Millipore, Burlington, Massachusetts, USA), α-Diap2 [33], α-Drice [34], α-HA (clone 3F10, #11867423001, Roche, Basel, Switzerland), α-V5 (Clone SV5-Pk1, #MCA1360, Bio-Rad, Hercules, California, USA), α-Myc (#M4439, Sigma), α-phosphohistone H3 (PHH-3) (Ser10, #9701, Cell Signalling Technology, Danvers, Massachusetts, USA) and α-Actin (C-11, sc-1615, Santa Cruz Biotechnology, Dallas, Texas, USA).

### Purification of GST-TUBE-fusion protein

GST-TUBE expression was induced in *E. coli* BL21 by the addition of 0.2 mM IPTG to an overnight culture of bacteria in LB medium at 18 °C. Bacteria were lysed by sonication in lysis buffer containing 50 mM Tris (pH 8.5), 150 mM NaCl, 3 mM DTT, 0.5 mM phenylmethylsulfonyl fluoride and 0.2 mg/ml lysozyme. The lysate was added to a column with Glutathione Sepharose™ 4B (GE Healthcare, Chicago, Illinois, USA) and then washed with wash buffer containing 50 mM Tris (pH 8.5) and 150 mM NaCl. GST-TUBE was eluted in 50 mM Tris (pH 8.5), 150 mM NaCl, 10% glycerol, 3 mM DTT and 50 mM glutathione. The proteins were concentrated from the eluate using Amicon® Ultra-4 30 K centrifugal filter devices (Merck Millipore, Burlington, Massachusetts, USA).

### Purification of ubiquitin conjugates from cells and fly lysates

Cells were lysed in a buffer containing 20 mM NaH_2_PO_4_, 20 mM Na_2_HPO_4_ 1% NP-40 and 2 mM EDTA, and fly lysates in buffer containing 50 mM Tris (pH 7.5), 150 mM NaCl, 1% Triton X-100, 1 mM EDTA and 10% glycerol, supplemented with 1 mM DTT, 5 mM NEM, Pierce™ Protease and Phosphatase Inhibitor, 5 mM chloroacetamide and 1% SDS. Lysates were sonicated, diluted to 0.1% SDS, and cleared before incubation with Glutathione Sepharose™ 4B (GE Healthcare) and GST-TUBE for a minimum of 2 h under rotation at 4 °C. The beads were washed four times with ice cold PBS-0.1% Tween-20 and eluted using Laemmli sample buffer.

### Lysis of whole flies or fly organs for western blotting

Ten adult *Drosophila* flies, or twelve dissected intestines or carcasses from adult female flies, were homogenized and lysed 10 min on ice in a buffer containing 50 mM Tris (pH 7.5), 150 mM NaCl, 1% Triton X-100, 1 mM EDTA and 10% Glycerol. The lysates were cleared before the addition of Laemmli sample buffer.

### Quantitative RT-PCR (qPCR)

Ten *Drosophila* adult flies or transfected *Drosophila* S2 cells were homogenised using QIAshredder (QIAGEN) and total RNA was extracted with RNeasy Mini Kit (QIAGEN) according to the manufacturer’s protocol. cDNA was synthesised with iScript cDNA synthesis kit (Bio-Rad) according to the manufacturer’s protocol. qPCR was performed using SensiFAST™ SYBR Hi-ROX Kit (Bioline, London, UK). *rp49* was used as a housekeeping gene for ΔCt calculations. The following gene-specific primers were used to amplify cDNA: *Diptericin* (5′-ACCGCAGTACCCACTCAATC-3′, 5′-ACTTTCCAGCTCGGTTCTGA-3′), *Drosocin* (5′-CGTTTTCCTGCTGCTTGC-3′, 5′-GGCAGCTTGAGTCAGGTGAT-3′), *rp49* (5′-GACGCTTCAAGGGACAGTATCTG-3′, 5′-AAACGCGGTTCTGCATGAG-3′).

### Immunofluorescence of *Drosophila* intestines

Intestines from three or more female adult flies were dissected in PBS and fixed for 10 min in 4% paraformaldehyde. Samples were permeabilised with PBS-0.1% Triton X-100, 1 h at room temperature, washed with PBS and incubated over night at 4 °C with primary antibody PHH-3 (Ser10, #9701, Cell Signalling Technology) at 1:1000. After washing, the intestines were incubated 2 h at room temperature with secondary antibody Alexa Fluor 488 donkey anti-rabbit IgG (#A21206, Invitrogen) at 1:600. Both primary and secondary antibodies were diluted in PBS and 1% bovine serum albumin. DNA was stained with DAPI (4′,6-diamidino-2-phenylindole) (Invitrogen). After washing with PBS, the samples were mounted using Mowiol (Sigma). Counting of PHH-3 positive cells was done using fluorescent microscopy (Zeiss Axiovert-200M microscope, Oberkochen, Germany)

### X-gal staining of *Drosophila* intestines and carcasses

Intestines or carcasses from three or more adult female flies were dissected in PBS and fixed 15 min at room temperature with PBS containing 0.4% glutaraldehyde and 1 mM MgCl_2._ The samples were washed with PBS and incubated with fresh staining solution containing 5 mg/ml X-gal, 5 mM potassium ferrocyanide trihydrate, 5 mM potassium ferrocyanide crystalline and 2 mg/ml MgCl_2_ in PBS 1 h at 37 °C. After washing with PBS, the samples were mounted in Mowiol and imaged with bright field microscopy (Leica, Wetzlar, Germany).

### Fluorometric measurement of caspase-3/7 activity and WST-1 assay

For fluorometric measurement of caspase-3/7 activity in flies, three dissected intestines or carcasses from adult female flies were lysed in buffer containing 50 mM Tris (pH 7.5), 150 mM NaCl, 1% Triton X-100, 10% glycerol, 1 mM EDTA and Pierce™ Protease Inhibitor. The lysate was cleared at 12,000 rpm for 10 min at 4 °C and protein concentration adjusted with Bradford assay (Bio-Rad). For fluorometric measurement of caspase-3/7 activity in *Drosophila* S2 cells, 2 × 10^6^ cells were transfected as described above. The caspase-3/7 activity of the cells and fly lysates was analysed using Apo-ONE^®^ Homogenous Caspase-3/7 Assay (Promega, Madison, Wisconsin, USA) according to the manufacturer’s protocol. Fluorescence was measured at 499/521 with the plate reader HIDEX sense (HIDEX, Turku, Finland). To measure cell viability, the WST-1 agent (Roche) was added to transfected cells in a ratio of 1:10. The absorbance was measured at 450 nm with the HIDEX plate reader after a 2-h incubation.

### Sequencing of the 16S rRNA gene

**Fig. 1:**
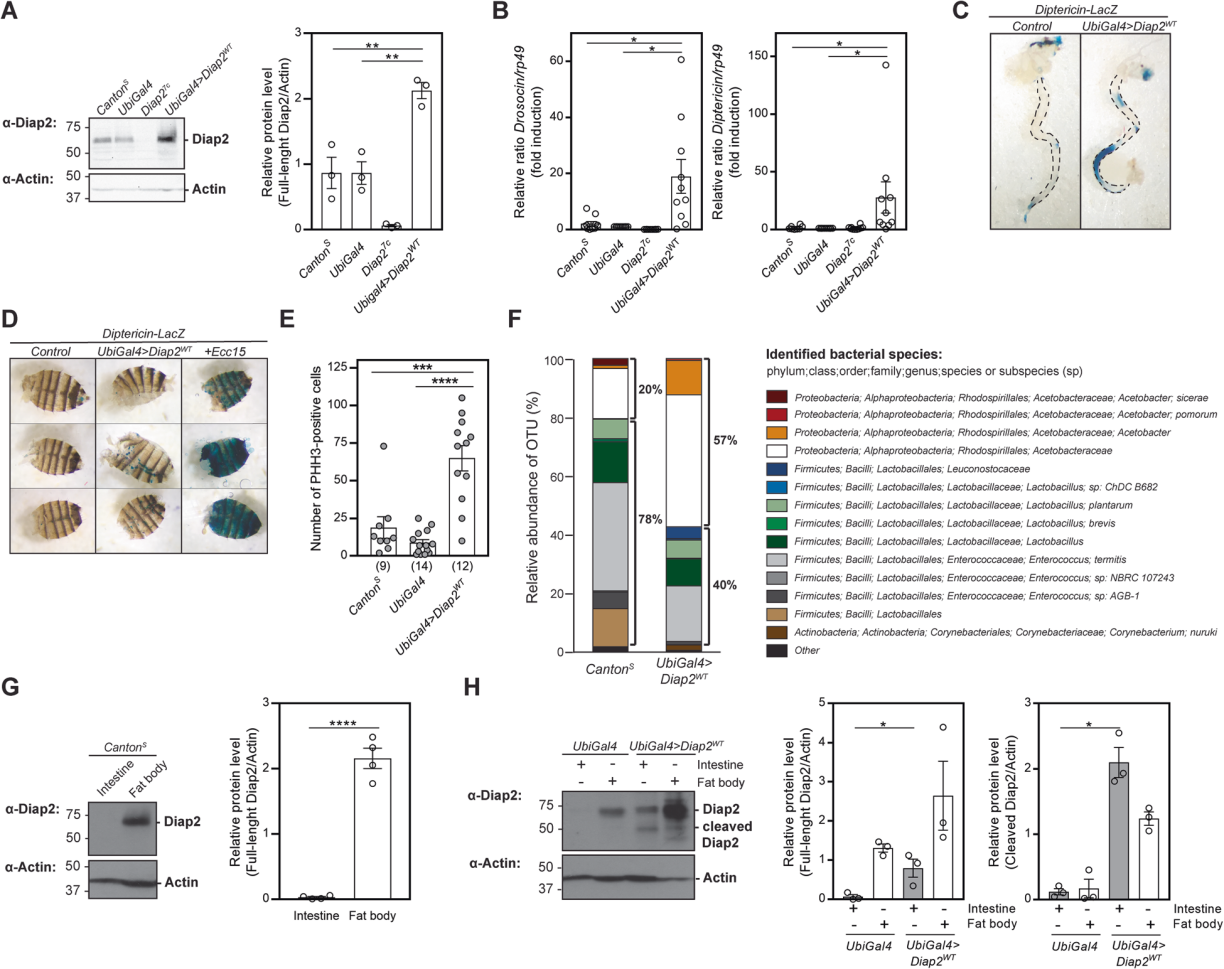
Diap2 induces chronic inflammation and hyperplasia in the fly intestine. **A** Whole fly lysates from *Canton*^*S*^, *UbiGal4*, *Diap2*^*7c*^ and *UbiGal4;UAS-Diap2*^*WT*^ flies were analysed by western blotting with α-Diap2 and α-Actin antibodies, and the relative protein level of Diap2 was quantified, *n* = 3. **B** Relative *Droso*c*in* and *Diptericin* mRNA levels analysed with qPCR in adult *Canton*^*S*^, *UbiGal4*, *Diap2*^*7c*^ and *UbiGal4;UAS-Diap2*^*WT*^ flies, *n* = 10. Adult female intestines (**C**) and carcasses (**D**) from *Diptericin-LacZ* and *UbiGal4;UAS-Diap2*^*WT*^*/Diptericin-LacZ* flies were dissected and stained for β-galactosidase activity, *n* = 3. The last lane in **D** shows a positive control for fat body activation induced by septic infection with *Ecc15*. **E** Intestines from adult *Canton*^*S*^, *UbiGal4* and *UbiGal4;UAS-Diap2*^*WT*^ flies were dissected and stained for phosphohistone H3 (PHH-3), and the number of positive cells were counted. The number of intestines analysed is indicated in brackets. **F** Bacterial 16S rRNA metagenomics analysis of the 1V-3V region in *Canton*^*S*^ and *UbiGal4;UAS-Diap2*^*WT*^ flies. Colours indicate identified operational taxonomic units (OTUs). Black brackets indicate proportions of *Proteobacteria* and *Firmicutes*, *n* = 1. Intestines and fat bodies from adult female *Canton*^*S*^ (**G**) or *UbiGal4* and *UbiGal4;UAS-Diap2*^*WT*^ (**H**) flies were dissected and lysed, and analysed by western blotting with α-Diap2 and α-Actin antibodies, *n* ≥ 3. The relative protein levels of full-length Diap2 (**G**) and full-length and cleaved Diap2 (**H**) were quantified. Data represent mean ± SEM. *p < 0.05, **p < 0.01, ***p < 0.001, ****p < 0.0001.

Genomic DNA was isolated from 40 adult flies using a modified protocol for the QIAamp DNA mini kit (QIAGEN) [35]. Flies were surface sterilized by vortexing them twice in 2% active hypochlorite and sterile H_2_O. The efficiency of the washes was confirmed by 16S PCR of water from the last wash step. Flies were homogenized in lysis buffer containing 20 mM Tris, pH 8.0, 2 mM EDTA, 1.2% Triton X-100 and 20 mg/ml lysozyme, and incubated 90 min at 37 °C. Overall, 200 µl AL buffer (QIAamp DNA mini kit) with 20 µl proteinase K were added and the lysate was incubated 90 min at 56 °C. Subsequent extraction was performed according to the manufacturer’s protocol. Amplification and Illumina MiSeq sequencing of the V1-V3 region of the 16S rRNA gene, as well as selection of operational taxonomic units (OTUs) and taxonomy assignment of OTUs was done using Eurofins Genomics InView Microbiome Profiling 3.0 service. In order to ensure similar *Wolbachia* status of both control and mutat fly line, the *Wolbachia* positive or negative state of the sequenced fly lines was tested by PCR with the *Wolbachia* specific primers: 5′-GWATTACCGCGGCKGCTG-3′ and 5′-AGAGTTTGATCCTGGCTCAG-3′ prior to sequencing. *Canton*^*S*^ and *UbiGal4* > *Diap2*^*WT*^ flies were positive for *Wolbachia*. The proportion of *Wolbachia* species have been omitted in Fig. 1F for easier comparison of bacterial species residing in the gut lumen.

### Statistical analysis

Results from survival assays were analysed by two-way analysis of variance with Tukey’s post hoc test for 95% confidence intervals and results from qPCR by two-tailed Student’s *t*-test on the ΔCt value, graphs depict relative fold induction of the target gene compared to a normalised control sample. The number of PHH-3 positive cells, relative fluorescence units, and the relative protein expression from western blots measured with ImageJ, were analysed by two-tailed Student’s *t*-test. In comparison to normalised control values, the Mann–Whitney U test was applied. In figures, ns stands for *p* > 0.05, *p < 0.05, **p < 0.01, ***p < 0.001 and ****p < 0.0001. Error bars in figures specify ±SEM from the indicated number of independent experiments. Experiments were repeated at least three times.

## Results

### Diap2 induces chronic inflammation and hyperplasia in the fly intestine

The *Drosophila* IAP protein Diap2 is required for inducing the Relish-dependent Imd pathway and mounting immune responses upon gram-negative bacterial infection, both locally in the epithelial layers of the gut and trachea, and upon systemic infections in the cells of the fat body [10, 12]. However, it is still unknown how the activity of Diap2 is regulated during basal conditions and upon pathogen-induced infections. To investigate how Diap2-mediated inflammatory responses are regulated, we analysed the impact of Diap2 on inflammatory NF-κB activation. For this purpose, we made transgenic flies, in which expression of Diap2 was induced via the UAS-Gal4 system (Fig. 1A). Analysis of the expression of the Relish target genes *Drosocin* and *Diptericin* showed that these AMPs are induced in the Diap2-expressing flies compared to *Canton*^*S*^ and *Ubiquitin-Gal4* (*UbiGal4*) control flies (Fig. 1B). To investigate the origin of Diap2-induced inflammation, we used the *Diptericin-LacZ* reporter system to compare *Diptericin* expression in the two major immune organs of the fly, the gut and the fat body [17]. We found that transgenic expression of Diap2 leads to a spontaneous induction of *Diptericin* in the *Drosophila* midgut, but not in the fat body (Fig. 1C, D).

Intestinal inflammation is associated with midgut hyperplasia and proliferating cells can be detected in *Drosophila* by staining the proliferation marker PHH-3 [36]. We found that Diap2-expressing flies have a significantly increased number of proliferating cells in the midgut compared to control flies (Fig. 1E). Another factor associated with chronic inflammation is dysbiosis of the gut microbiome. When profiling the bacterial composition of wild type and Diap2 transgenic flies by 16S sequencing, we found the Diap2-expressing flies to have a higher ratio of *Proteobacteria* to *Firmicutes* compared to control flies (Fig. 1F), a notion that has been associated with increased gut inflammation in both flies and humans [37, 38].

Finally, as Diap2 overexpression leads to different inflammatory phenotypes in gut and fat body, we compared the endogenous expression of Diap2 in these organs of *Canton*^*S*^ wild type flies. We were able to detect Diap2 in fat body samples, but not in the gut (Fig. 1G). However, when examining the protein levels of Diap2 in Diap2 transgenic flies, we were able to detect, in addition to full-lenght Diap2 in the fat body, both a full-length and a truncated version of Diap2 in the intestine (Fig. 1H), indicating that increased expression of Diap2 induces inflammation only in the intestine.

### Drice restrains intestinal activation of the Imd pathway and gut hyperplasia

**Fig. 2:**
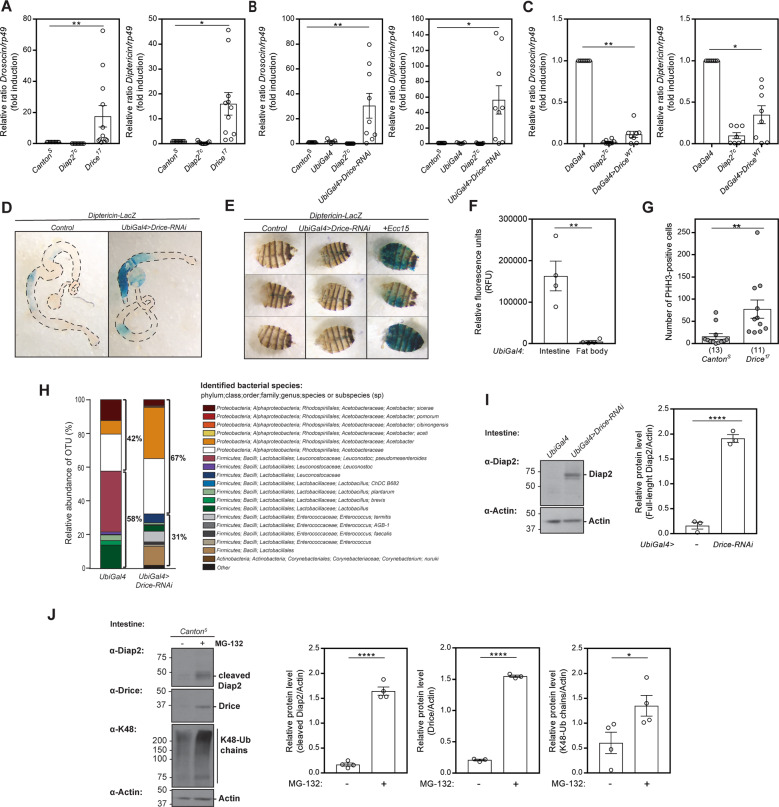
Drice restrains intestinal activation of the Imd pathway and gut hyperplasia. Relative *Drosocin* and *Diptericin* mRNA levels analysed with qPCR in adult *Canton*^*S*^, *Diap2*^*7c*^ and *Drice*^*17*^ flies (**A**), in *Canton*^*S*^, *UbiGal4*, *Diap2*^*7c*^ and *UbiGal4;UAS-Drice-RNAi* (**B**) flies or in *DaGal4*, *Diap2*^*7c*^ and *UAS-Drice*^*WT*^*;DaGal4* flies (**C**), *n* ≥ 8. Adult female intestines (**D**) and carcasses (**E**) from *Diptericin-LacZ* and *UbiGal4;UAS-Drice-RNAi/Diptericin-LacZ* flies were dissected and stained for β-galactosidase activity, *n* = 3. The last lane in **E** shows a positive control for fat body activation induced by septic infection with *Ecc15*. **F** Adult female intestines or fat bodies from *UbiGal4* flies were dissected and lysed, and the caspase-3/7 activity was assessed after addition of Apo-ONE reagent by measuring fluorescence at 499/521 nm, *n* = 4. **G** Intestines from adult *Canton*^*S*^ and *Drice*^*17*^ flies were dissected and stained for phosphohistone H3 (PHH-3), and the PHH-3 positive cells were counted. The number of intestines analysed is indicated in brackets. **H** Bacterial 16S rRNA metagenomics analysis of the 1V-3V region in *UbiGal4* and *UbiGal4;Drice-RNAi* flies. Colours indicate identified operational taxonomic units (OTUs). Black brackets indicate proportions of *Proteobacteria* and *Firmicutes*, *n* = 1. **I** Intestines from adult *UbiGal4* and *UbiGal4;UAS-Drice-RNAi* were dissected and lysed, and analysed by western blotting with α-Diap2 and α-Actin antibodies, *n* = 3, and the relative protein level of full-length Diap2 was quantified. **J** Adult *Canton*^*S*^ flies were fed 50 μM MG-132, their intestines were dissected and analysed by western blotting with α-Diap2, α-Drice, α-K48 and α-Actin antibodies, *n* ≥ 3. The relative protein levels of cleaved Diap2, Drice and K48-Ub chains were quantified. Data represent mean ± SEM, *p < 0.05, **p < 0.01, ****p < 0.0001.

Caspases belong to a family of conserved cysteine-dependent endoproteases that cleave their substrates after specific aspartic residues [39]. The *Drosophila* effector caspase Drice is one of the key inducers of apoptosis in the fly [40]. Diap2 and Drice have been shown to interact and form a stable complex, wherein Diap2 inhibits the activity of Drice by binding to, and ubiquitinating the caspase. This inhibition is mechanism-based, requiring Drice activity, and results in cleavage of Diap2 [41]. As the truncated form of Diap2 expressed in the intestine of Diap2 transgenic flies corresponds in size to the Drice-cleaved form of Diap2, we wanted to investigate a possible role for Drice in the regulation of Diap2-mediated inflammatory signalling. For this purpose, we measured the expression of NF-κB target genes in *Drice*^*17*^ mutant flies and in *Drice-RNAi* flies. *Drice*^*17*^ flies encode an unstable form of the Drice protein, reported to express less than 5% of the levels of Drice in wild-type flies [42], and neither *Drice*^*17*^ nor *Drice-RNAi* flies have detectable expression of the protein in our study (Fig. S1A, B). We detected a significantly higher expression of the Relish target genes *Drosocin* and *Diptericin* during basal conditions in whole fly lysates from both *Drice*^*17*^ (Fig. 2A) and *Drice-RNAi* flies (Fig. 2B) and, accordingly, a lower basal *Drosocin* and *Diptericin* expression in flies overexpressing Drice (Fig. 2C), suggesting that Drice acts as a negative regulator of Imd signalling.

To investigate if the Drice-mediated regulation of the Imd pathway is tissue-specific, we examined *Diptericin* expression in the gut and fat body of *Drice-RNAi* flies carrying the *Diptericin-LacZ* reporter gene. Similarly to Diap2 overexpression, loss of *Drice* induced *Diptericin* expression in the midgut but not in the fat body (Fig. 2D, E). Interestingly, active Drice, assessed by measuring DEVD-activity, was also detected only in dissected fly guts and not in the fat body (Fig. 2F). As in the Diap2-expressing flies, we found *Drice*^*17*^ mutant flies to have a significantly increased number of proliferating cells in the midgut, compared to *Canton*^*S*^ control flies (Fig. 2G). Further, *Drice-RNAi* flies have a higher ratio of *Proteobacteria* to *Firmicutes* compared to *UbiGal4* driver flies (Fig. 2H). Taken together, these results show that Drice is a negative regulator of Imd signalling, needed for maintaining gut homeostasis.

Finally, Diap2 is stabilised in the intestine of *Drice-RNAi* flies, suggesting that Drice and the formation of the Drice-Diap2 complex regulate the levels of Diap2 in the *Drosophila* intestine (Fig. 2I). In addition, we found that inhibiting the proteasome by feeding flies MG-132 leads to a stabilization of Drice, the cleaved form of Diap2, as well as an increase in K48-linked chains in the intestine (Fig. 2J). This indicates that formation of the Drice-Diap2 complex induces degradation of both proteins in a manner that is preceded by cleavage of Diap2.

### The catalytic activity of Drice is required for Imd pathway regulation in the intestine

**Fig. 3:**
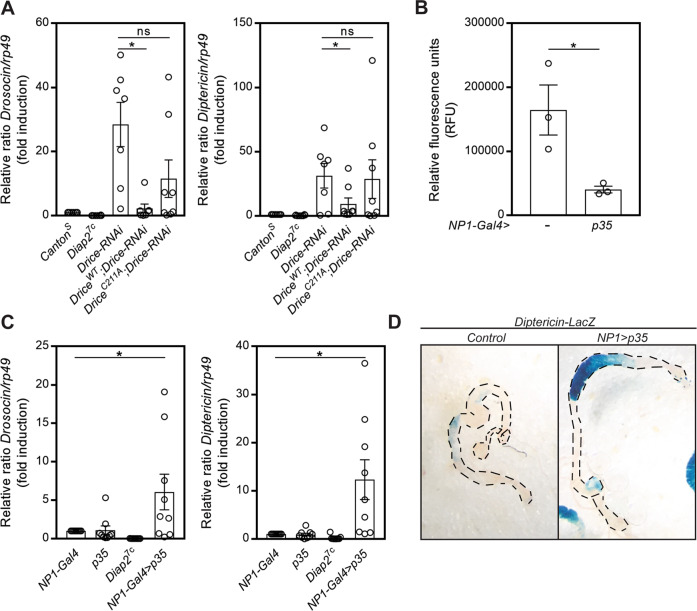
The catalytic activity of Drice is required for Imd pathway regulation in the intestine. **A** Relative *Drosocin* and *Diptericin* mRNA levels analysed with qPCR in adult *Canton*^*S*^, *Diap2*^*7c*^, *UbiGal4*;*UAS*-*Drice-RNAi*, *UAS*-*Drice*^*WT*^*/UbiGal4;UAS-Drice-RNAi* and *UAS*-*Drice*^*C211A*^*/UbiGal4;UAS-Drice-RNAi* flies, *n* = 7. **B** Adult female intestines from *NP1-Gal4* and *NP1-Gal4;UAS-p35* flies were dissected and lysed, and the caspase-3/7 activity was assessed after addition of Apo-ONE reagent by measuring fluorescence at 499/521 nm, *n* = 3. **C** Relative *Drosocin* and *Diptericin* mRNA levels analysed with qPCR in adult *NP1-Gal4*, *UAS-p35*, *Diap2*^*7c*^ and *NP1-Gal4;UAS-p35* flies, *n* = 9. **D** Adult female intestines from *DaGal4, Diptericin-LacZ* and *NP1-Gal4;UAS-p35/Diptericin-LacZ* flies were dissected and stained for β-galactosidase activity, *n* = 3. Data represent mean ± SEM, *p < 0.05.

To investigate whether the regulation of Diap2 is reliant of the catalytic activity of Drice, we generated transgenic flies expressing Drice^WT^ or the catalytically inactive Drice^C211A^ mutant. While *Drice-RNAi* eliminated the endogenous Drice, the transgenic expressed Drice remained high in a *Drice-RNAi* background (Fig. S2). When we analysed the expression of the Relish target genes *Drosocin* and *Diptericin* in whole fly lysates during basal conditions, we found that only Drice^WT^, but not Drice^C211A^, is able to restrain the AMP expression induced by loss of Drice (Fig. 3A).

To verify that Drice is the caspase regulating Diap2, we expressed the effector caspase inhibitor p35 in the intestinal epithelial cells with the enterocyte-specific driver *NP1-Gal4*. When cleaved by a caspase, the viral caspase inhibitor p35 acts as a suicide substrate by trapping the catalytic machinery of the caspase via a covalent thioacyl linkage [43]. p35 has been shown to inhibit the *Drosophila* effector caspases Drice and Dcp-1 [44], which both recognise the DEVD amino acid sequence [34, 40]. However, Drice is shown to be the only *Drosophila* effector caspase to interact with Diap2 [45]. We found that expression of p35 in intestinal enterocytes led to reduced effector caspase activity (Fig. 3B) and increased expression of the Relish target genes *Drosocin* and *Diptericin* (Fig. 3C). Likewise, a local induction of *Diptericin* expression in the intestine was detected in *Diptericin-LacZ* flies expressing the p35 caspase inhibitor (Fig. 3D). Our results indicate, thus, that the catalytic cysteine and, thereby, the covalent bond formed between Diap2 and Drice is needed for Drice to regulate the activity of the Imd pathway, and that p35, by trapping the catalytic cysteine of Drice, interferes with the ability of Drice to restrain Imd signalling. Although the initiator caspases Dredd and Dronc have been shown to interact with Diap2 [46, 47], neither Dredd nor Dronc is inhibited by p35 [44, 48], suggesting that Drice alone inhibits Diap2-mediated activation of Relish target genes by interacting with Diap2.

### Drice regulates Diap2 levels and ubiquitination of Dredd and Kenny

**Fig. 4:**
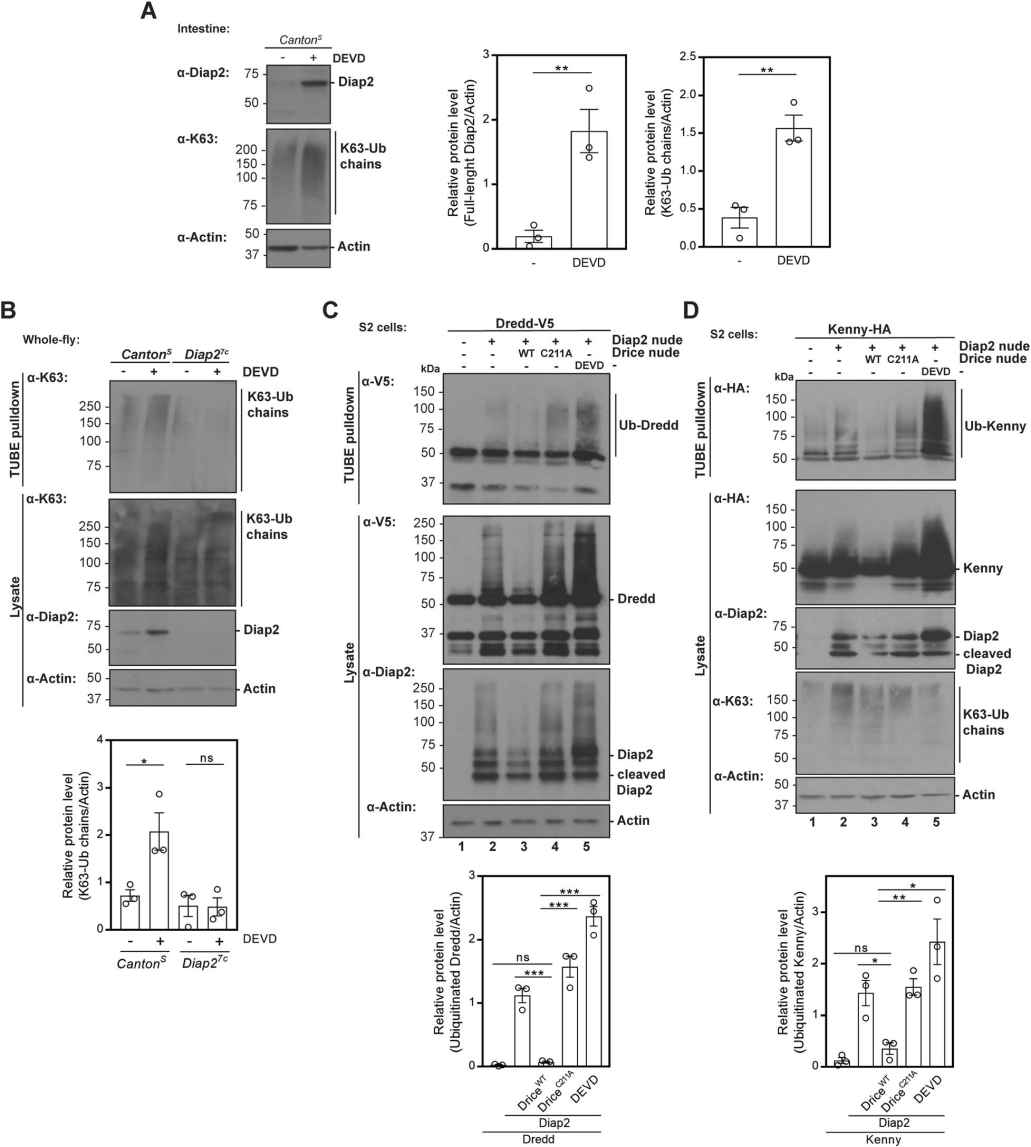
Drice regulates Diap2 levels and ubiquitination of Dredd and Kenny. **A** Adult *Canton*^*S*^ flies were fed 50 μM Z-DEVD-FMK, their intestines were dissected, lysed and subjected to western blotting analysis with α-Diap2, α-K63 and α-Actin antibodies, *n* = 3. The relative protein levels of full-length Diap2 and K63-linked ubiquitin chains were quantified. **B** *Canton*^*S*^ and *Diap2*^*7c*^ flies were fed 50 μM Z-DEVD-FMK and lysed. Ubiquitin chains were isolated with GST-TUBE under denaturing conditions and the samples were analysed by western blotting with α-K63, α-Diap2, α-Drice and α-Actin antibodies, *n* = 3. The relative protein level of K63-Ub chains was quantified. *Drosophila* S2 cells were transfected with empty vector, Diap2^WT^, Drice^WT^, Drice^C211A^, V5-tagged Dredd (**C**) or with HA-tagged Kenny (**D**), where after the cells were treated with 20 µM Z-DEVD-FMK. Ubiquitin chains were isolated with GST-TUBE at denaturing conditions and the samples were analysed by western blotting with α-V5, α-HA, α-Diap2, α-K63 and α-Actin antibodies, *n* = 3. The relative protein level of ubiquitinated Dredd or Kenny was quantified. Data represent mean ± SEM, *p < 0.05, **p < 0.01, ***p < 0.001.

Diap2-mediated K63-Ub of the Imd pathway components Imd and Dredd has been shown to be required for Imd signalling, additionally the *Drosophila* NEMO, IKKγ or Kenny, has recently been identified as a target of K63-Ub mediated by Diap2 [25–27]. To investigate if inhibition of Drice in the intestine affects Diap2-mediated ubiquitination, we fed flies with the cell-permeable caspase-3 inhibitor Z-DEVD-FMK. Indeed, a local intestinal inhibition of Drice led to the accumulation of full-length Diap2 in gut samples (Fig. 4A) and to a Diap2-dependent increase in K63-linked ubiquitin chains pulled down with recombinant GST-TUBE from whole fly lysates (Fig. 4B). To investigate further whether Drice interferes with the ability of Diap2 to ubiquitinate Dredd and Kenny, we co-transfected S2 cells with Diap2^WT^ and Dredd-V5 or Kenny-HA, while inducing Drice activity by overexpressing Drice, or interefering with the catalytic activity by overexpressing Drice^C211A^ or treating with the inhibitor Z-DEVD-FMK (Fig. S3A, B). Importantly, by measuring the activity of mitochondrial dehydrogenases with the WST-1 assay, we verified that cell viability was not affected by overexpression of Drice^WT^ or Drice^C211A^ in S2 cells (Fig. S3C). Ubiquitin chains were pulled down with recombinant GST-TUBE from cell lysates under denaturing conditions. When overexpressing Diap2^WT^ alone, we could detect ubiquitin smears on both Dredd and Kenny (Fig. 4C, D, lanes 2). Co-expression with Drice^WT^ reduced the amount of Diap2, and also the ubiquitination of Dredd and Kenny (Fig. 4C, D, lanes 3). Likewise, overexpression of catalytically inactive Drice^C211A^, and treatment with Z-DEVD-FMK that stabilized full-length Diap2, resulted in enhanced ubiquitination of Dredd and Kenny (Fig. 4C, D, lanes 4 and 5). This indicates that the levels of Diap2 can be regulated by exogenous manipulation of Drice activity in S2 cells, restraining the ability of overexpressed Diap2 to ubiquitinate the Imd pathway inducers Dredd and Kenny.

### Drice does not restrain pathogen-induced inflammatory signalling

**Fig. 5:**
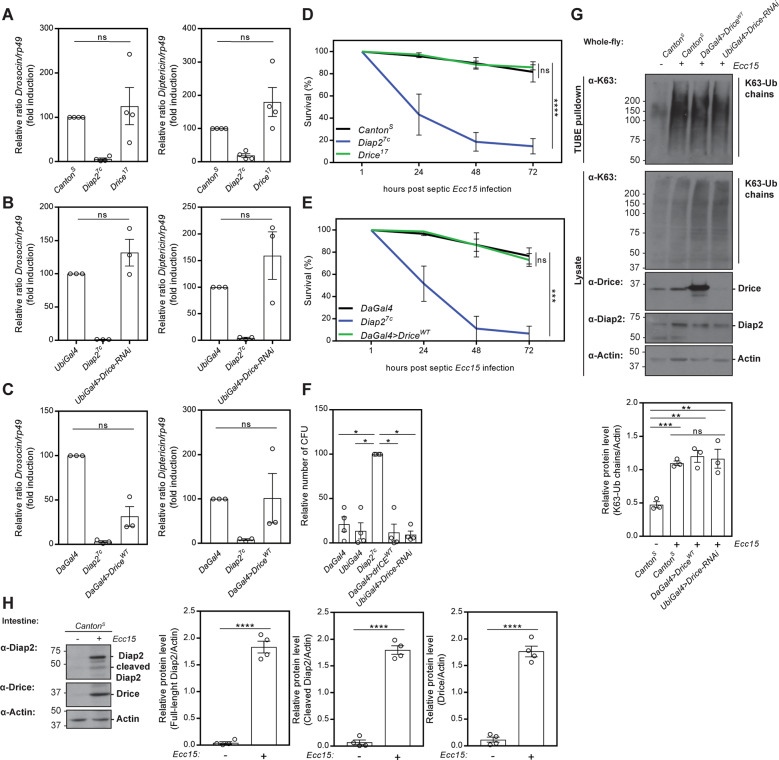
Drice does not restrain pathogen-induced inflammatory signalling. Relative *Drosocin* and *Diptericin* mRNA levels analysed with qPCR in *Canton*^*S*^, *Diap2*^*7c*^, *Drice*^*17*^ (**A**) *UbiGal4;UAS-Drice-RNAi* (**B**) and in *DaGal4*, *Diap2*^*7c*^ and *UAS-Drice*^*WT*^*;DaGal4* (**C**) flies 5 h after septic infection with the gram-negative bacteria *Ecc15*, *n* ≥ 3. Adult *Canton*^*S*^, *Diap2*^*7c*^ and *Drice*^*17*^ (**D**) or *DaGal4*, *Diap2*^*7c*^ and *UAS-Drice*^*WT*^*;DaGal4* (**E**) flies were subjected to septic injury with *Ecc15* and their survival was monitored over time, *n* = 3. **F** *DaGal4*, *UbiGal4*, *Diap2*^*7c*^, *UAS-Drice*^*WT*^*;DaGal4* and *UbiGal4* > *Drice-RNAi* flies were infected by feeding with *E. coli* for 24 h and the bacterial load was assessed by counting colony-forming units (CFU), *n* = 4. **G** The induction of K63-Ub chains was analysed in *Canton*^*S*^, *UAS-Drice*^*WT*^*;DaGal4* and *UbiGal4* > *Drice-RNAi* flies 5 h after septic infection with *Ecc15*. Ubiquitin chains were isolated with GST-TUBE under denaturing conditions and samples analysed by western blotting with α-K63, α-Diap2, α-Drice and α-Actin antibodies, *n* = 3. The relative protein level of K63-Ub chains was quantified. **H** Adult flies were infected by feeding *Ecc15* 16 h, their guts were dissected, lysed and used in western blot analysis with α-Drice, α-Diap2 and α-Actin antibodies, *n* = 4. The relative protein levels of full-length Diap2, cleaved Diap2 and Drice were quantified. Data represent mean ± SEM. *p < 0.05, **p < 0.01, ***p < 0.001, ****p < 0.0001.

As Drice seems to restrain Diap2 from inducing inflammatory signal transduction, the immune response upon septic pathogen-infection was tested in Drice-overexpressing and in Drice-mutant flies. Interestingly, *Canton*^*S*^ control flies, *Drice*^*17*^, *Drice-RNAi* flies, as well as flies overexpressing wild-type Drice (Fig. 5A–C) showed a similar induction of *Drosocin* and *Diptericin* 5 h after septic infection with *Ecc15*. Furthermore, when monitoring the survival of flies, neither control flies, *Drice*^*17*^ flies, nor *Drice*-overexpressing flies, succumb to septic infection with *Ecc15* (Fig. 5D, E). To investigate if the Drice-mediated regulation of Diap2 affected local immune signalling in the intestine, we analysed the response to ingested pathogen. By performing a bacterial colony count after a 24-h feeding on *E. coli*, we found that both Drice-overexpressing flies and *Drice-RNAi* flies were able to fend off pathogens ingested with food, like control flies (Fig. 5F). In addition, K63-Ub was induced to similar level in Drice-overexpressing flies, *Drice-RNAi* flies and wild type *Canton*^*S*^ flies upon septic infection with *Ecc15* (Fig. 5G). Likewise, no reduction of Diap2 levels nor a significant decrease in Diap2-mediated ubiquitination of Dredd and Kenny could be detected upon Drice^WT^ overexpression after activating the Imd pathway by overexpression of PGRP-LCx in S2 cells (Fig. S4A, B). These results suggest that Drice does not inhibit the induction of Diap2-mediated ubiquitination and activation of Imd signalling during infection. Supporting this, pathogenic infection coincides with stabilization of full-length Diap2, cleaved Diap2 and Drice in the gut, indicating that the presence of pathogens leads to the disruption of the intestinal Drice-Diap2 complex, freeing both proteins (Fig. 5H). This would indicate that Drice is able to regulate only basal immune responses.

While Diap2 cleavage is a consequence of interaction with Drice, we wanted to examine if cleavage of Diap2, and, thereby, separation of the BIR1 domain, also reduces its activity in the Imd pathway. However, both flies expressing Diap2^WT^ and flies expressing constitutively cleaved Diap2^Δ100^ in a *Diap2*^*7c*^ mutant background were able to induce AMPs and survive upon septic infections, and clear pathogens locally in the intestine similarly to control flies (Fig. S4C, D, E). These results indicate that the Drice-cleaved form of Diap2, indeed still harbouring BIR2 and BIR3, mediating Imd and Dredd binding [25, 26], a UBA and a RING domain, is a functional mediator of Imd signalling. Hence, we suggest that the role of Drice is to induce cleavage-mediated degradation of Diap2 in the absence of infection.

### Drice restrains inflammatory signalling induced by the resident microbiome

**Fig. 6:**
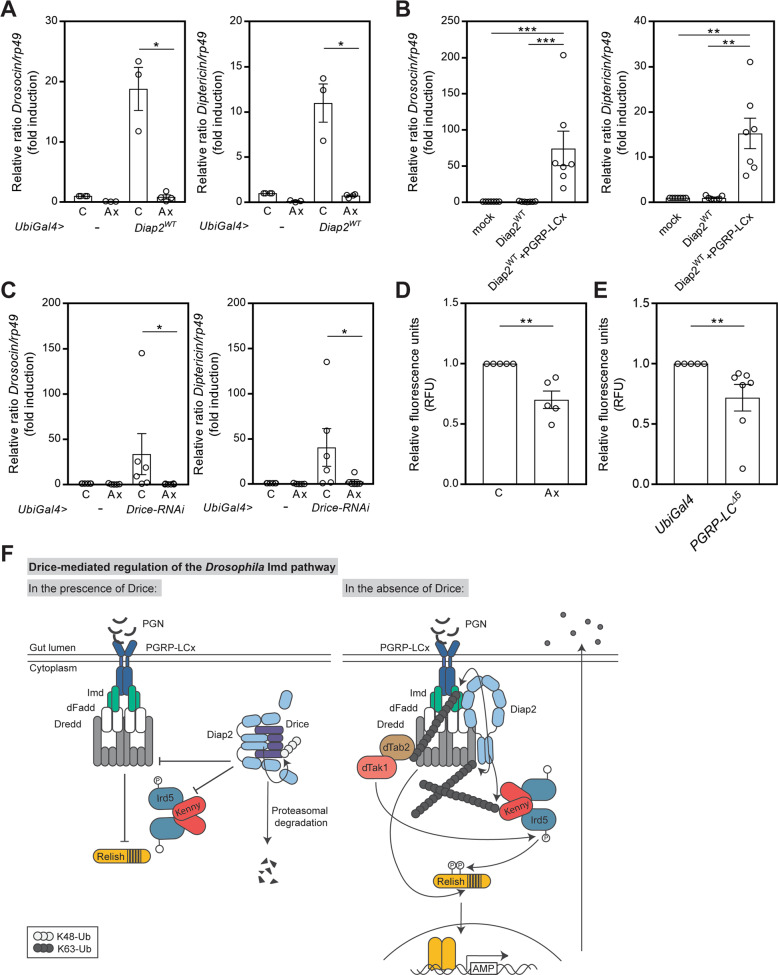
Drice restrains inflammatory signalling induced by the resident microbiome. **A** Relative *Drosocin* and *Diptericin* mRNA levels were analysed by qPCR in conventionally reared (C) or axenic (Ax) adult *UbiGal4* and *UbiGal4;UAS-Diap2*^*WT*^ flies, *n* = 3. **B** Relative *Drosocin* and *Diptericin* mRNA levels were analysed by qPCR from *Drosophila* S2 cells transfected with empty vector, Diap2^WT^ and PGRP-LCx, *n* = 7. **C **Relative *Drosocin* and *Diptericin* mRNA levels analysed with qPCR in conventionally reared (C) or axenic (Ax) adult *UbiGal4* and *UbiGal4;UAS-Drice-RNAi* flies, *n* = 6. **D** Caspase-3/7 activity in adult female guts of conventionally reared (C) or axenic (Ax) *yellow white* control flies was analysed by measuring fluorescence at 499/521 nm after addition of Apo-ONE reagent, *n* = 5. **E** Caspase-3/7 activity in adult female guts of *PGRP-LC*^*Δ5*^ mutant flies and *UbiGal4* control flies was analysed by measuring fluorescence at 499/521 nm after addition of Apo-ONE reagent, *n* = 5. Data represent ∓ SEM, *p < 0.05, **p < 0.01, ***p < 0.001. **F** Proposed model for Drice-mediated regulation of Imd signalling. PGN from the cell wall of commensal bacteria induces a low activation of the PGRP-LCx receptor, leading to the recruitment of Imd, dFadd and Dredd to the receptor complex. To regulate NF-κB activation, active Drice binds to Diap2 to form the Drice-Diap2 complex, which is subsequently targeted for proteasomal degradation. As a consequence, ubiquitination of Kenny and Dredd is decreased. In the absence of Drice, uncontrolled activation of intestinal Imd-signalling leads to excessive levels of AMPs in the gut lumen and a disturbed gut homeostasis. Unrestrained Diap2 drives Imd sigalling by ubiquitinating Imd, Dredd and Kenny. Ubiquitination of Imd enables recruitment of the dTab2-dTak1 complex, and dTak1-mediated activation of Ird5 by phosphorylation. Ird5 in turn phosphorylates Relish. Ubiquitination-dependent activation of Dredd and Dredd-mediated cleavage of Relish precedes translocation of the Relish dimer to the nucleus and subsequent activation of Relish target genes.

As the bacterial presence is constant in the gut, and the fat body only encounters bacteria during a systemic infection [49], we hypothesized that the commensal microbiome activates Diap2-mediated Imd-signalling, which in the absence of Drice, leads to an excessive inflammatory response. To eliminate the commensal intestinal microbiome, we reared flies under axenic conditions. As expected, the expression of *Drosocin* and *Diptericin* was no longer elevated in axenic Diap2^WT^ expressing flies (Fig. 6A). Likewise, overexpression of Diap2^WT^ in S2 cells was not able to induce AMP expression in the absence of receptor activation (Fig. 6B). The increased expression of *Drosocin* and *Diptericin* detected in *UbiGal4* > *Drice-RNAi* flies was, as in Diap2^WT^ expressing flies, significantly decreased by rearing the flies under axenic conditions (Fig. 6C), indicating a link between commensal bacteria and the requirement for Drice-mediated regulation of Imd signalling.

Interestingly, Diap2 is undetectable in the guts of axenic wild-type flies (Fig. S5), indicating that degradation of the Diap2-Drice complex is continuous also in a bacteria-free environment. This is further supported by a reduction in caspase activity in the intestines of both wild-type axenic and PGRP-LCx mutant flies, compared to conventionally reared flies or control flies, respectively (Fig. 6D, E). Taken together, we propose that commensal bacteria trigger the formation of an initial PGRP-LCx receptor complex, competing for Diap2-recruitment to further activate the Imd pathway. The inflammatory response is counteracted by Drice binding to, and forming a complex with Diap2 that is subsequently targeted for degradation. Hence, Drice interferes with the ability of Diap2 to induce downstream signalling and NF-κB target gene activation under basal conditions (Fig. 6F).

## Discussion

Caspases were first identified as regulators of apoptosis, but their regulatory functions now extend to other cellular processes, such as immune signalling and development. Dysregulation of caspases has been implied in tumorigenesis, autoimmunity, autoinflammation and infectious pathologies [39, 50, 51]. Caspases are known to be regulated by IAP proteins, however, caspase-mediated regulation of IAP proteins during inflammatory signalling has not been established before. Here, we report that the *Drosophila* caspase Drice functions as a negative regulator of intestinal immune responses regulated via the *Drosophila* NF-κB Relish. While Drice does not seem to impact pathogen-induced immune signalling, it restrains responses induced by commensal bacteria by binding Diap2, leading to the degradation of both proteins. This indicates that Drice halts NF-κB signalling by trapping Diap2 to a degradation complex during basal conditions, and that a pathogenic infection leads to complex disruption and freeing of a functional Diap2. The constitutive interaction between Diap2 and Drice in the intestinal epithelia may also contribute to the regulation of caspase activation to avoid apoptosis-induced cell proliferation [52].

We suggest that the low activation of PGRP-LCx induced by commensal bacteria is sufficient to induce Diap2-mediated Relish activation in the absence of a brake. In the intestinal epithelia, the brake is provided by the effector caspase Drice, halting unwanted inflammatory responses. Interestingly, while Drice transcription is moderate or low in most *Drosophila* tissues during normal conditions, including the fat body, transcription of Drice is high in the adult *Drosophila* intestine [53]. This indicates a specific need for continuous expression of Drice to overcome the constant Drice turnover, protecting from unwanted Diap2-induced inflammatory responses in the intestinal cells. As Diap2 has been shown to ubiquitinate Drice [41], and itself [26], we speculate that formation of a Diap2-Drice complex induces Diap2-mediated K48-linked ubiquitination of both proteins, targeting them for proteasomal degradation. Further characterization of this mechanism and identification of the E2 ubiquitin-conjugating enzymes involved is, however, needed.

While Drice and Diap2 are targeted for degradation during normal conditions, they both are stabilised upon activation of the PGRP-LCx receptor. We suggest that the receptor stimulation leads to disruption of a pre-existing Drice-Diap2 complex, as also the Drice-cleaved form of Diap2 is stabilised. However, it cannot be excluded that Diap2 is released from Drice due to competition by the activated receptor complex. Regardless, we suggest that the released Diap2 is redirected for ubiquitination of new target substrates during infection. A similar IAP-induced shift in target ubiquitination upon receptor activation has been shown in non-canonical NF-κB signalling, where cIAPs switch from degradation-inducing ubiquitination of the NF-κB-inducing kinase (NIK) to ubiquitination of TRAF2/3 upon receptor activation, releasing NIK to activate downstream signalling [54].

While the Drice-Diap2 complex formation leads to the degradation of both proteins, Diap2 cleavage, separating the BIR1 domain is also a consequence of this interaction [41]. The Diap2 homologue XIAP is a key regulator of NOD2-induced inflammatory signalling in the mammalian intestine, shown to induce the pathway by ubiquitinating RIPK2 [55, 56]. Interestingly, a nonsense mutation E99X in XIAP that introduces a stop codon after the BIR1 domain was found in an early onset Crohn’s disease patient. This mutation induced a severe and selective defect in intestinal NOD signalling, without affecting immune signalling in T cells and peripheral blood mono-nuclear cells [57]. This suggests that a separated BIR1 domain is associated with impaired NF-κB signalling also in mammalian intestinal cells. The BIR1 domain has been shown to regulate XIAP-mediated NF-κB signalling, by bringing TAB1 and XIAP together, leading to activation of TAK1 [58]. Hence, it will be interesting to study if the separated BIR1 of Diap2 mediates NF-κB responses via, the yet unidentified, *Drosophila* TAB1-homologue in the epithelial cells of the fly intestine.

Intestinal epithelial cells coexist with commensal bacteria and need to develop tolerance to pattern recognition to allow for healthy host–microbe interactions. However, these epithelial cells also need to maintain responsiveness to foodborne pathogens. Hence, proper regulation of NF-κB signalling is crucial to avoid deregulated immune responses and chronic inflammation deleterious for the host [59]. We propose that IAP regulation may provide a mechanism of restraining unwanted inflammatory responses in cells of epithelial tissues exposed to non-pathogenic microbiota. Interestingly, the caspase-mediated restriction in IAP activity, we describe, does not affect pathogen-induced responses. Hence, it may allow for specific regulation of malfunctional epithelial responses in chronic inflammatory disease.

## Supplementary information

Supplementary figures

Supplementary figure legends

## Data Availability

All data generated or analysed during this study are included in this published article and in its supplementary information file.
