# Supplementary material for: Drice restrains Diap2-mediated inflammatory signalling and intestinal inflammation
Source: Cell Death Differ. 2021 Jul 14;29(1):28–39. doi: 10.1038/s41418-021-00832-w (PMC8738736; doi:10.1038/s41418-021-00832-w)

Supplementary figures

Drice restrains Diap2-mediated inflammatory signalling and intestinal inflammation

Christa Kietz, Aravind K Mohan, Vilma Pollari, Ida-Emma Tuominen, Paulo S Ribeiro, Pascal Meier, Annika Meinander

Kietz et al. Supplementary Figure 1

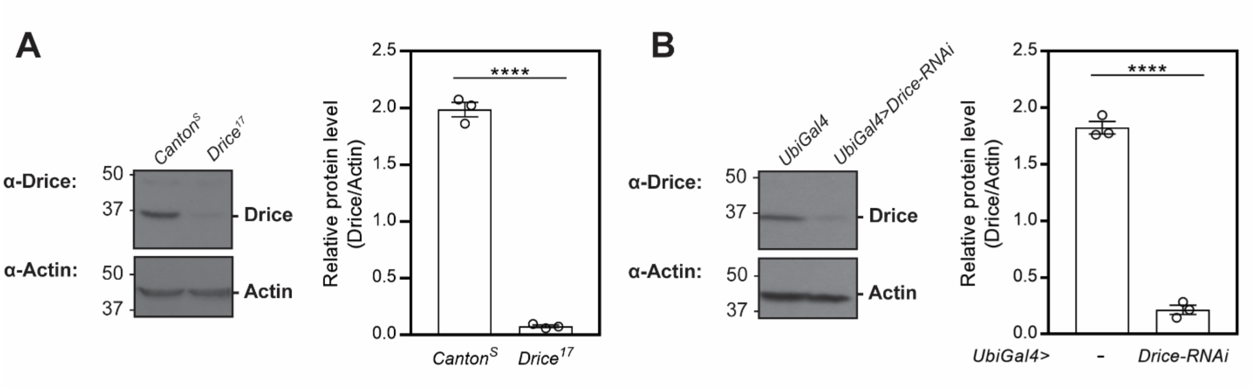

Kietz et al. Supplementary Figure 2

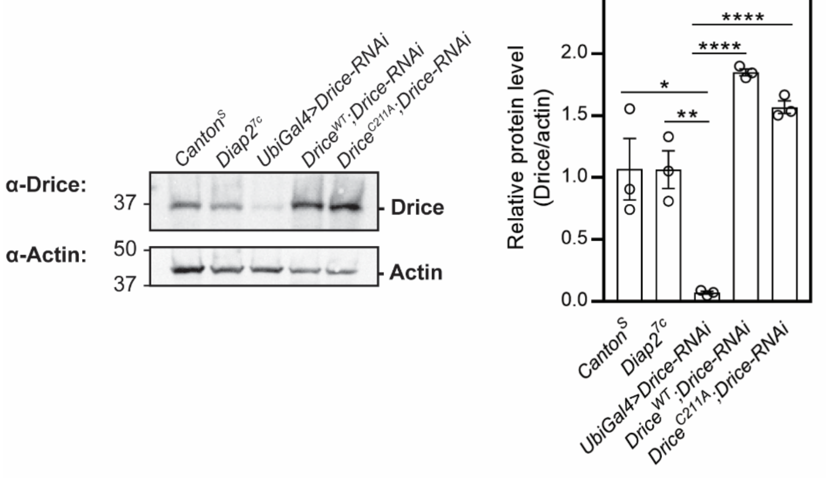

Kietz et al. Supplementary Figure 3

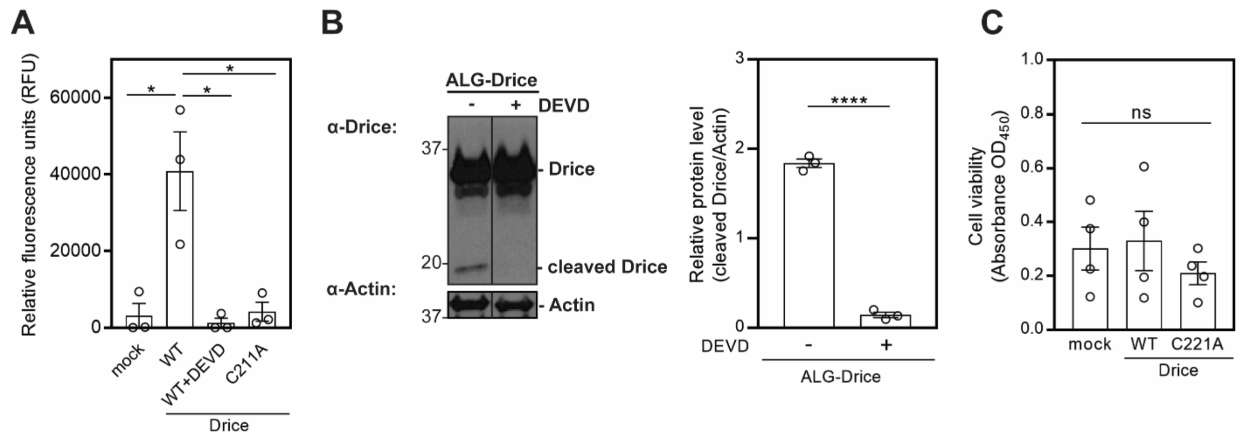

Kietz et al. Supplementary Figure 4

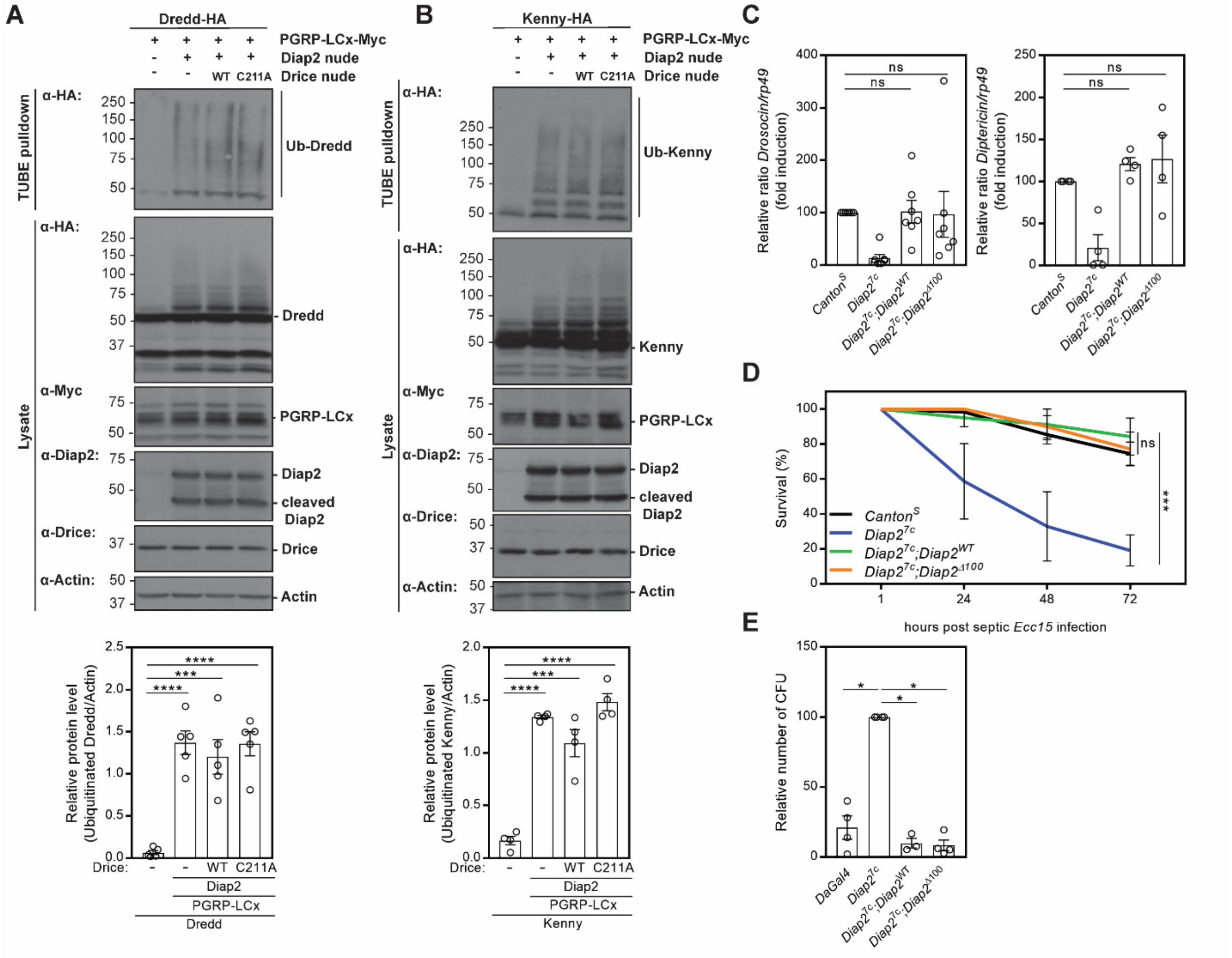

Kietz et al. Supplementary Figure 5

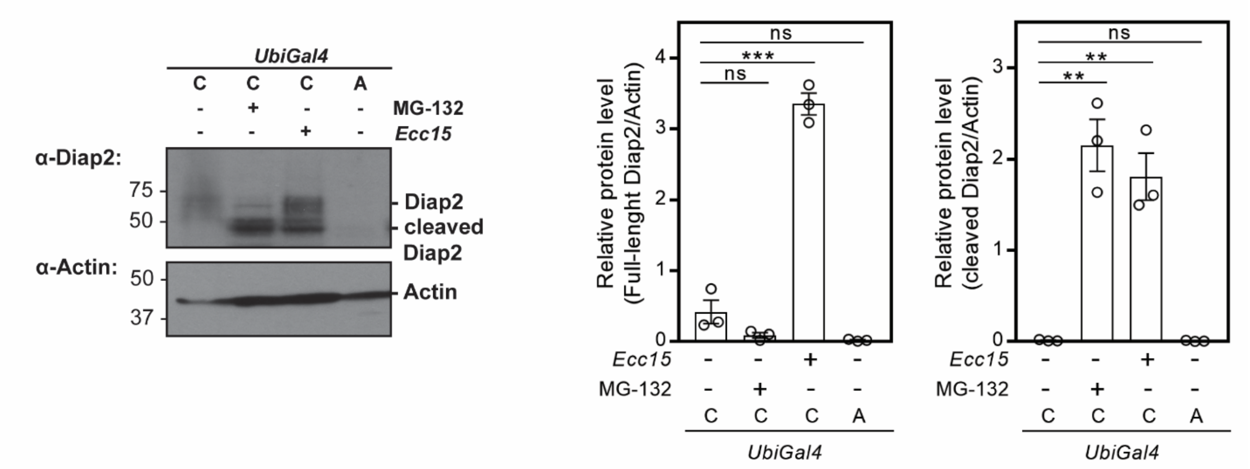

Supplement: Supplementary file 1 — Supplementary figures [file 41418_2021_832_MOESM1_ESM.pdf]
