# Supplementary material for: Drice restrains Diap2-mediated inflammatory signalling and intestinal inflammation
Source: Cell Death Differ. 2021 Jul 14;29(1):28–39. doi: 10.1038/s41418-021-00832-w (PMC8738736; doi:10.1038/s41418-021-00832-w)
Supplement: Supplementary file 2 — Supplementary figure legends [file 41418_2021_832_MOESM2_ESM.docx]

**Supplementary** **figure legends**

**Drice restrains Diap2-mediated inflammatory signalling and intestinal inflammation**

Christa Kietz, Aravind K Mohan, Vilma Pollari, Ida-Emma Tuominen, Paulo S Ribeiro, Pascal Meier, Annika Meinander

**Supplementary Figure 1.** Drice protein levels in Drice mutants A, B) Whole fly lysates from *Canton^S^* and *Drice^17^* (A) or *UbiGal4* and *UbiGal4;UAS-Drice-RNAi* (B) flies were analysed by Western blotting with α-Drice and α-Actin antibodies, n = 3. The relative protein level of Drice was quantified. Data represent mean ± SEM and **** p < 0.0001.

**Supplementary Figure 2.** Drice protein level in flies expressing Drice^WT^ and Drice^C211A^ in *Drice-RNAi* background. Whole fly lysates from *Canton^S^*, *Diap2^7c^*, *UbiGal4*;*UAS*-*Drice-RNAi*, *UAS*-*Drice^WT^/UbiGal4;UAS-Drice-RNAi* and *UAS*-*Drice^C211A^/UbiGal4;UAS-Drice-RNAi* were analysed by Western blotting with α-Drice and α-Actin antibodies, and the relative protein level of Drice quantified, n = 3. Data represent mean ± SEM. * p < 0.05, ** p < 0.01, **** p < 0.0001.

**Supplementary Figure 3.** Verification of Drice inhibition by DEVD-treatment, and of cell viability after Drice overexpression in S2-cells. A) *Drosophila* S2-cells were transfected with empty vector, Drice^WT^ or Drice^C211A^, where after the cells were treated with 20 µM Z-DEVD-FMK 16 h. The caspase-3/7 activity was analysed by adding Apo-ONE reagent to plated cells and measuring fluorescence at 499/521 nm, n = 3. B) *Drosophila* S2-cells were transfected with ALG-Drice and treated with Z-DEVD-FMK for 16 h. Cells were lysed and Drice cleavage was analysed by Western blotting with α-Drice and α-Actin antibodies, n = 3. The relative protein level of cleaved Drice was quantified. C) S2-cells were transfected with empty vector, Drice^WT^ or Drice^C211A^ and the cell viability assessed by addition of WST-1 reagent and measurement of absorbance at 450 nm, n = 4. Data represent mean ± SEM. ns stands for non-significant, * p < 0.05, **** p < 0.0001.

**Supplementary Figure 4.** Ubiquitination of Dredd and Kenny upon PGRP-LCx overexpression, and immune response in *Diap2^Δ100^* expressing flies. A, B**)** *Drosophila* S2 cells were transfected with empty vector, Diap2^WT^, PGRP-LCx-Myc, Drice^WT^, Drice^C211A^ and HA-tagged Dredd (A) or HA-tagged Kenny (B). Ubiquitin chains were isolated with GST-TUBE at denaturing conditions and the samples were analysed by Western blotting with α-HA, α-Diap2, α-Drice, α-Myc and α-Actin antibodies, n ≥ 4. The relative protein levels of ubiquitinated Dredd or Kenny were quantified. C) Relative *Drosocin* and *Diptericin* mRNA levels analysed with qPCR in adult *Canton^S^*, *Diap2^7c^*, *Diap2^7c^;UAS-Diap2^WT^/DaGal4* and in *Diap2^7c^;UAS-Diap2^Δ100^/DaGal4* flies 5 h after septic infection with *Ecc15*, n ≥ 4. D) Adult *Canton^S^*, *Diap2^7c^*, *Diap2^7c^;UAS-Diap2^WT^/DaGal4* and *Diap2^7c^;UAS-Diap2^Δ100^/DaGal4* flies were subjected to septic injury with *Ecc15* and their survival was monitored over time, n = 4. E) *DaGal4, Diap2^7c^*, *Diap2^7c^;UAS-Diap2^WT^/DaGal4 Diap2^7c^;UAS-Diap2^Δ100^/DaGal4* flies were infected by feeding with *E. coli* for 24 h and the bacterial load was assessed by counting colony-forming units (CFU), n = 4. The same *DaGal4* and *Diap2^7c^* controls were used as in Figure 5F. Data represent mean ± SEM. ns stands for non-significant, * p < 0.05, *** p < 0.001, **** p < 0.0001.

**Supplementary Figure 5.** Diap2 protein levels in axenic flies. The intestines from adult female conventionally reared (C) *UbiGal4* flies treated with 50 μM MG-132 or orally infected with *Ecc15*, and of axenic (A) *UbiGal4* flies were dissected and lysed and analysed by Western blotting with α-Diap2 and α-Actin antibodies, n = 3. The relative protein levels of full-length and cleaved Diap2 were quantified. Data represent mean ± SEM. ns stands for non-significant and ** p < 0.01, *** p < 0.001.
